# Supplementary material for: Artificial intelligence guided conformational mining of intrinsically disordered proteins
Source: Commun Biol. 2022 Jun 20;5:610. doi: 10.1038/s42003-022-03562-y (PMC9209487; doi:10.1038/s42003-022-03562-y)
Supplement: Supplementary file 1 — Supplementary Information [file 42003_2022_3562_MOESM1_ESM.pdf]

Artificial Intelligence Guided Conformational Mining of Intrinsically Disordered Proteins

Aayush Gupta[1], Souvik Dey[1], Alan Hicks, and Huan-Xiang Zhou[1,2,*]

[1]Department of Chemistry and [2]Department of Physics, University of Illinois at Chicago, Chicago, Illinois 60607, USA

*e-mail: hzhou43@uic.edu

Supplementary Information

Supplementary Note 1

Instead of Cartesian coordinates, rotationally invariant properties such as dihedral angles or distance matrices could be used as the input to autoencoders. We tested such models. Training using 30% of the MD run1 data for Aβ40, with backbone dihedral angles (three per residue) as input and a latent-space dimension of 200, we obtained a reconstruction RMSD (C_α only) of 11.78 Å (calculated on the 100-fold diluted test set of 980 conformations). In comparison, the reconstruction C_α RMSD using Cartesian coordinates of the same training set and the same latent-space dimension was only 3.28 Å. The reconstruction results (RMSDs calculated on heavy atoms) for the latter Cartesian model are reported in Fig. S2.

The reconstruction RMSE of the dihedral model was 35°, but the error of any individual dihedral angle could be as large as 100° (calculated from the largest error among the individual dihedral angles in each test conformation, averaged over the test set). A dihedral error this large in the middle of the IDP chain produces RMSDs of 12.3 ± 3.7 Å (calculated on 10 test conformations), because the dihedral angle affects the Cartesian coordinates of all the residues in the second half of the chain.

Autoencoders using a distance matrix as input had a similar problem. For example, using C_α-C_α distances of residue i to residues $i + 1$, $i + 2$, $i + 3$, $i + 4$ as input, the reconstruction C_α RMSD for Aβ40 was 11.13 Å. An i to $i + 3$ C_α-C_α distance corresponds to a virtual dihedral angle defined by the C_α atoms of residues i, $i + 1$, $i + 2$, and $i + 3$. Therefore the high reconstruction RMSD of the distance-matrix model can in particular be attributed to errors in the i to $i + 3$ C_α-C_α distance. The reconstruction RMSE of the distance-matrix model was 1.1 Å for the i to $i + 3$ C_α-C_α distance, and the corresponding mean largest error was 3.0 Å.

Here are some details of the models and data analysis when dihedral angles or distance matrices were used as input. The autoencoder architecture was the same as described for the case where Cartesian coordinates were the input, with a dimension of 200 for the latent space. Again the loss function was the binary cross-entropy. For dihedral angles, the number of input and output neurons was $3N_{\mathrm{res}} - 3$ (117 for Aβ40); for distance matrices, the number of input and output neurons was $4N_{\mathrm{res}} - 10$ (150 for Aβ40). The conversion from Cartesian coordinates (backbone heavy atoms only) to dihedral angles was done using the BAT (bond-angle-torsion) module [1] in the MDAnalysis package [2]. The back conversion was also done using the BAT module, with each bond or bond angle fixed at the average value of the training set. For distance matrices, the back conversion to Cartesian coordinates (C_α atoms only) was done in a Fortran code, with C_α Cartesian coordinates of residues i, $i + 1$, $i + 2$, and $i + 3$ calculated from the i to $i + 1$, $i +1$

to $i + 2$, $i + 2$ to $i + 3$, i to $i + 2$, $i + 1$ to $i + 3$, and i to $i + 3$ distances. This procedure generated two possible positions for the $i + 3$ C_α (mirror images of each other); the $i - 1$ to $i + 3$ distance was used to select one of the two image positions. That is, we selected the $i + 3$ C_α position with an $i - 1$ to $i + 3$ distance closer to the supplied $i - 1$ to $i + 3$ distance.

Supplementary Note 2

Figure S4 and Table S1 show that the training data in the latent space from MD run1 of ChiZ is not represented well by a single multivariate Gaussian. We explored the idea of representing the training data by a mixture of multivariate Gaussians. To that end, we first clustered the latent-space positions of the training set. The clustering method was k-means cluster and the distances between latent-space positions were the Euclidian distances. The number of clusters was set to 2, 4, or 8. We then used the mean vector and covariance matrix calculated on the points within each cluster to define a cluster-specific multivariate Gaussian. Lastly each cluster-specific Gaussian was assigned a weight proportional to the number of points in that cluster, and new points were sampled from all the cluster-specific Gaussians, with a particular Gaussian selected each time based its assigned weight. The new points were fed to the decoder to produce conformations in Cartesian coordinates.

We compared a generated set of conformations (at size 1×) against the 10-fold diluted training set and test set. The best-match RMSDs are listed below. Without clustering (i.e., a single Gaussians), the best-match RMSD with the training set was 6.06 Å; this RMSD decreases as the number of Gaussians increases, reaching 4.65 Å with 8 Gaussians. Thus a mixture of multivariate Gaussians indeed improved the representation of the training data in the latent space. However, the degree of match with the test set went in the opposite direction. The best-match RMSD increased steadily from 7.95 Å for a single Gaussian to 8.50 Å with 8 Gaussians. The mixture of multivariate Gaussians was apparently overfitting the training data, thereby compromising the ability to capture generic features shared by the test data.

# of Gaussians	Best-match RMSD with training set (Å)	Best-match RMSD with test set (Å)
1	6.06	7.95
2	5.71	8.05
4	5.26	8.22
8	4.65	8.50

Supplementary Note 3

We assessed the effect of increasing the training size on the accuracy of generated conformations in match with the test set. For MD run1 of each IDP, we included a growing percentage of conformations in the training set, from 10% to 70%. The best-match RMSDs with the corresponding test sets are listed below.

For Q15, as the training size increased from 10% to 70%, there was a slight improvement in prediction accuracy, with the best-match RMSD decreasing from 3.59 Å to 3.44 Å. We did not deem the small gain in accuracy (a mere 0.15 Å reduction in RMSD) at the cost of expanding the MD simulations by 7 times was justified, and hence we selected 10% as the training size for Q15. For Aβ40, a similar slight improvement in prediction accuracy was obtained, with the best-match RMSD plateauing at ~5.2 Å when the training size reached 50%, compared to 5.60 Å at the 20% training size. We chose 20% as the training size for Aβ40 as a comprise between accuracy and cost. For ChiZ, the best-match RMSD already reached plateau at the 30% training size, and that was our final selection for training size.

	Best-match RMSD (Å) at size 1×[b]		
Training size[a]	Q15	Aβ40	ChiZ
10%	**3.59**[c]	5.94	10.08
20%	3.52	**5.60**[c]	8.18
30%	3.50	5.47	**7.95**[c]
50%	3.48	5.15	8.23
70%	3.44	5.22	7.89

[a]Size expressed as percentage of the full dataset, from MD run1 of each protein.

[b]1× means that the generated set had the same size as the particular test set (e.g., 70% of the full dataset).

[c]Entries in bold are for the training sizes eventually selected in the autoencoders for the respective IDPs.

A practice popular in other applications of autoencoders is to randomize, or shuffle the data before separating into the training set and test set. For IDP conformations sampled from MD simulations, shuffling increases the similarity between the training and test sets, as conformations in the two sets become more likely to be near each other along the MD trajectory. When shuffling was applied, the best-match RMSDs were 3.05 Å for Q15 at 10% training size, 3.99 Å for Aβ40 at 20% training size, and 6.20 Å for ChiZ at 30% training size. Significant gains in prediction accuracy were indeed obtained upon data shuffling. However, doing so departs from our goal of achieving accuracy at the lowest cost of MD simulations.

We selected $0.75N_{res}$ as the latent-space dimension (=13 for Q15, 30 for Aβ40, and 48 for ChiZ), but also assessed the effect of the latent-space dimension on the prediction accuracy. The results are listed below. In short, while reducing the latent-space dimensions from the selected values by 10 resulted in deterioration in accuracy, increasing the dimensions by 10, 20, and 30 had little effect on the prediction accuracy. For Q15, a very large value, 200, for the latent-space dimension actually led to a slight increase in best-match RMSD (see also Fig. S6).

Latent-space dimension | best-match RMSD (Å) at size 1×[a]					
Q15		Aβ40		ChiZ	
3	3.82	20	5.88	38	8.29
**13**[b]	3.59	**30**[b]	5.60	**48**[b]	7.95
23	3.59	40	5.54	58	7.77
33	3.48	50	5.58	68	8.04
43	3.48	60	5.55	78	7.93
200	3.76				

[a]In each row under an IDP, the first entry is the latent-space dimension and the second entry is the best-match RMSD between the 100-fold diluted test set and the generated set at size 1×. Data are from MD run1 of each protein.
[b]Entries in bold are the latent-space dimensions eventually selected in the autoencoders for the respective IDPs.

Supplementary Note 4

The main results that we report for ChiZ were based on MD simulations using the AMBER14SB/TIP4PD force-field combination. To further demonstrate the robustness of conformational generation by autoencoders, we applied this approach to ChiZ conformations sampled from MD simulations using four other protein/water force-field combinations. For AMBER03ws/TIP4P2005, the amount of simulations was the same as for AMBER14SB/TIP4PD, i.e., 12 trajectories of 3 μs each. The autoencoder results for AMBER03ws/TIP4P2005 were very similar to those for AMBER14SB/TIP4PD. The reconstruction RMSDs for AMBER03ws/TIP4P2005 at a 30% training size were 7.1 ± 1.6 Å (mean ± standard deviation among 12 MD runs), comparable to the counterparts, 6.4 ± 1.0 Å, for AMBER14SB/TIP4PD. For generating new conformations, the best-match RMSDs at size 1× were 7.9 ± 1.3 Å for AMBER03ws/TIP4P2005; the corresponding result for AMBER14SB/TIP4PD run1 was 7.95 Å, right in the middle of the range of best-match RMSDs obtained from the 12 AMBER03ws/TIP4P2005 runs.

For each of the other three force-field combinations, we ran four trajectories of 0.5 μs each. Given the shorter trajectories, we used 70% of the conformations (25, 000 total) for training and 30% for testing. The reconstruction RMSDs were 7.2 Å for AMBER99SB-ILDN/TIP4PD, 5.9 Å for AMBER15IPQ/SPCEb, and 4.4 Å for CHARMM36m/TIP3Pm. For generating new conformations, the best-match RMSDs were each approximately 1 Å higher than the respective reconstruction RMSDs. Again, these ranges of RMSDs for conformational reconstruction and generation are comparable to those obtained using AMBER14SB/TIP4PD, thereby demonstrating the robustness of the autoencoder approach.

Supplementary Note 5

We tested autoencoders where the loss function was the mean square error (MSE) instead of the binary cross-entropy (BCE). The results, listed below, showed no significant differences between the two loss functions. Likewise, the particular conformation used for structural alignment before shifting and scaling the Cartesian coordinates for autoencoder input had no effect on the accuracy of generated conformations.

	Best-match RMSD (Å) at size 1×[a]		
	Q15	Aβ40	ChiZ
1st frame[b]; BCE[c]	3.59	5.60	7.95
1st frame[b]; MSE[d]	3.48	5.68	7.85
50000th frame[b], BCE[c]	3.51	5.56	7.81

[a]The data for training and testing were from MD run1 of each protein.

[b]The frame number used for structural alignment before shifting and scaling the coordinates.

[c]The loss function was binary cross-entropy (BCE).

[d]The loss function was mean square error (MSE).

Table S1. Kullback-Leibler divergence between histograms in two-dimensional spaces of the latent space

Fig. 3										
	0,3	5,6	7,10	12,13	15,20	21,22	23,25	0,27		
training | Gaussian	0.116	0.071	0.074	0.080	0.054	0.056	0.078	0.098		
test | Gaussian	0.376	0.079	0.110	0.122	0.731	0.335	0.209	0.543		
training | test	0.580	0.189	0.204	0.168	0.930	0.312	0.348	0.572		
Fig. 5a										
	4,6	9,14	15,16	24,26	28,30	34,36	38,39	44,47		
training | Gaussian	0.023	0.079	0.019	0.079	0.045	0.030	0.079	0.023		
test | Gaussian	0.042	0.081	0.097	0.089	0.027	0.037	0.073	0.032		
training | test	0.040	0.036	0.077	0.066	0.034	0.036	0.061	0.038		
Fig. S3										
	3,4	4,9	5,8	3,11	5,9	5,11	8,12	9,11	9,12	11,12
training | Gaussian	0.089	0.089	0.051	0.058	0.109	0.056	0.053	0.093	0.066	0.077
test | multivariate	0.063	0.068	0.051	0.051	0.071	0.055	0.054	0.063	0.068	0.055
training | test	0.084	0.097	0.078	0.136	0.097	0.105	0.073	0.101	0.080	0.105
Fig. S4										
	0,4	5,8	9,14	15,16	20,21	24,26	25,27	28,30	38,39	44,47
training | Gaussian	0.479	0.081	0.125	0.092	0.091	0.104	0.072	0.075	0.097	0.118
test | multivariate	0.767	0.215	0.257	0.702	0.116	0.193	0.266	0.223	0.267	0.343
training | test	0.362	0.188	0.452	0.424	0.181	0.319	0.282	0.311	0.378	0.294

Table S2. Diversity of test sets and similarity between training and test sets

	Pairwise RMSD (Å)[a] (dil test × dil test)	Best Match RMSD (Å)[b] (dil test × dil test)	Best Match RMSD (Å)[c] (dil test × train)
Q15 (10% run1)	6.98	3.71	3.96
Aβ40 (20% run1)	11.61	3.83	6.76
ChiZ (30% run1)	18.21	4.83	10.17
ChiZ (combined)[d]	19.23	8.62	8.47

[a]Average RMSD when each conformation is compared with all other conformations in the diluted test set.

[b]Average best-match RMSD of the diluted test set against other members of the same set.

[c]Average best-match RMSD of the diluted test set against the training set.

[d]Combined training or test set, each from combining the corresponding data from all of the 12 MD runs. The combined training set is diluted 10-fold before use, whereas the combined test set is diluted 1000-fold.

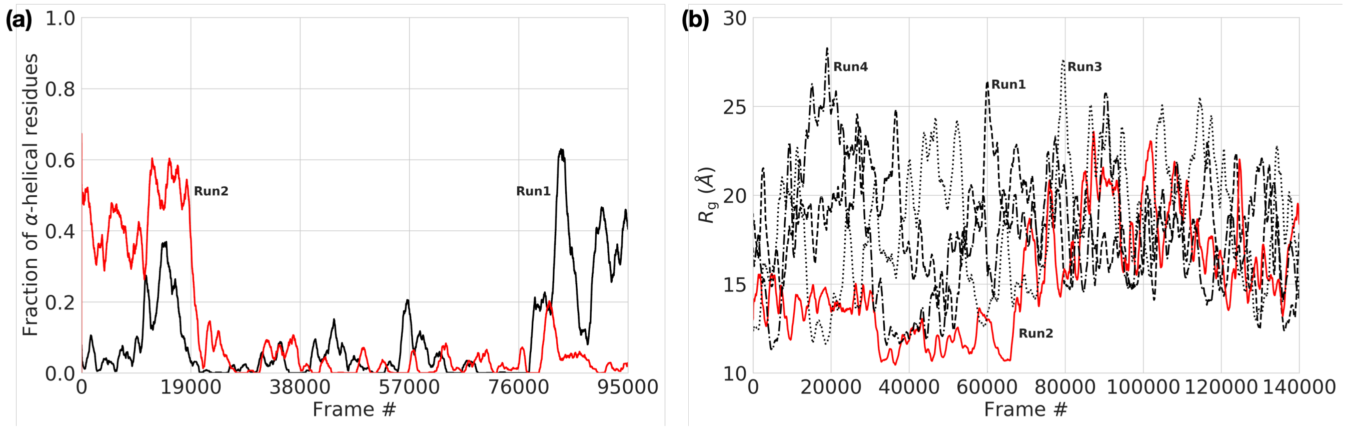

Figure S1. Conformational properties of Q15 and Aβ40 in MD simulations. (a) Fraction of α-helical residues in two replicate MD runs of Q15. Run1 started from a random-coil conformation whereas run2 started from an all α-helical conformation. These simulations were reported previously[3], where conformational sampling was enhanced by the replica exchange with solute tempering (REST) method[4,5]. (b) Radius of gyration of Aβ40 in four replicate runs. All the four runs started from disordered conformations, but run2 happened to stay relatively compact for the first half of the simulation. The curves were smoothed by running average with a window of 1000 frames (sampled at 10-ps intervals for Q15 and 20-ps intervals for Aβ40).

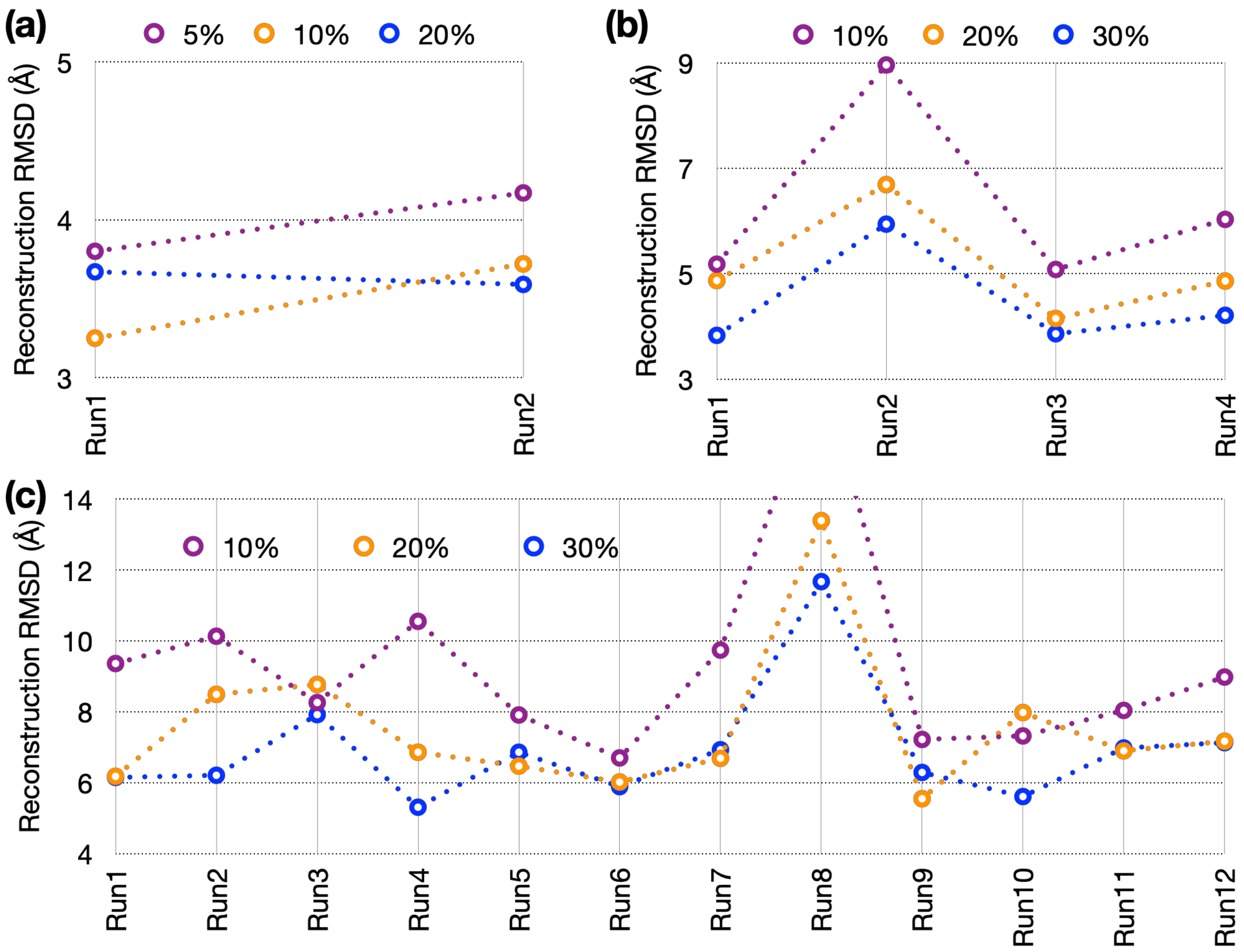

Figure S2. Reconstruction results when the dimension of the latent space is increased to 200. Average reconstruction RMSDs at different sizes of the training sets sampled from replicate MD runs. (a) Q15 at 5%, 10%, and 20% training sizes from two runs. (b) Aβ40 at 10%, 20%, and 30% training sizes from four runs. (c) ChiZ at 10%, 20%, and 30% training sizes from 12 runs.

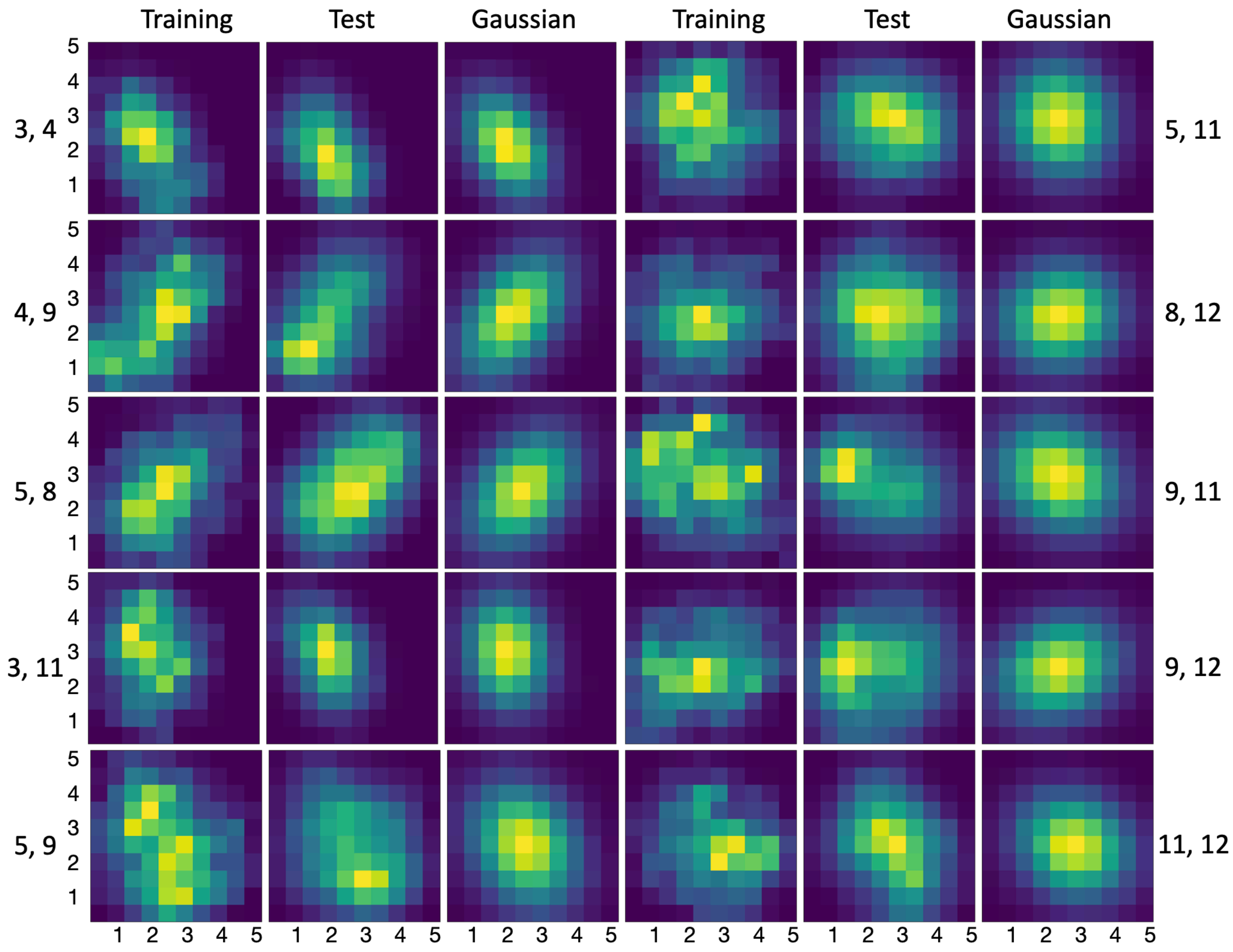

Figure S3. Histograms of Q15 in the latent space, calculated from training data, test data, and multivariate Gaussian. Histograms for pairs of encoder nonzero outputs from run1 are shown as heat maps, with yellow representing pixels with the highest counts and dark blue representing pixels with 0 count.

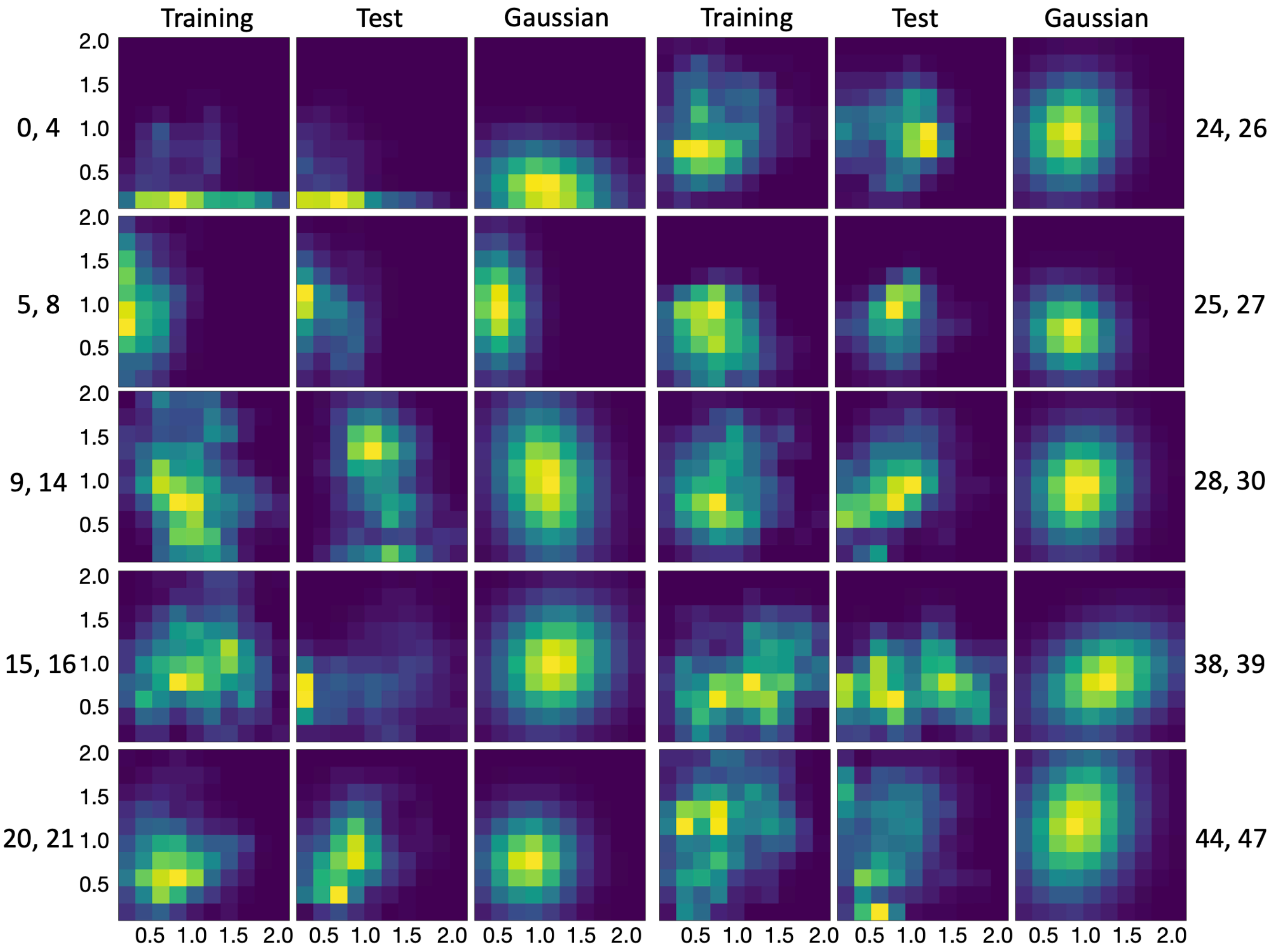

Figure S4. Histograms of ChiZ in the latent space, calculated from training data, test data, and multivariate Gaussian. Histograms for pairs of encoder nonzero outputs from run1 are shown as heat maps, with yellow representing pixels with the highest counts and dark blue representing pixels with 0 count.

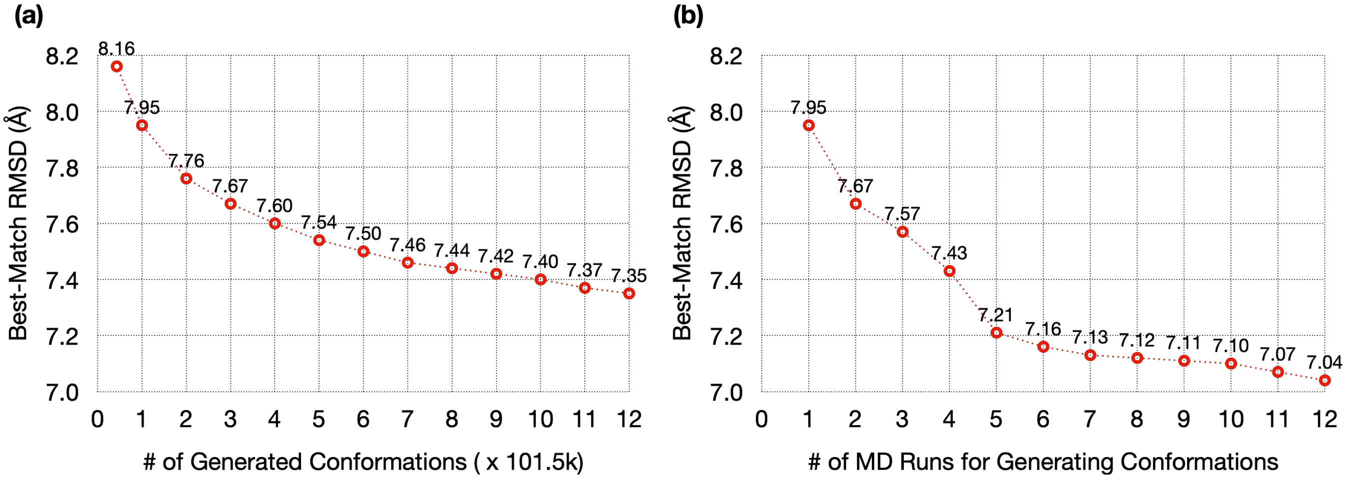

Figure S5. Results for autoencoder-generated conformations of ChiZ. The average best-match RMSDs of the 100-fold diluted test set of run1 are calculated against generated sets at different sizes. (a) Generated sets from run1, at sizes measured in multiples of the test size (= 101,500). (b) Generated sets pooled from run1 to run*i*, where *i* goes from 1 to 12. From each MD run, the generated set is at size 1×.

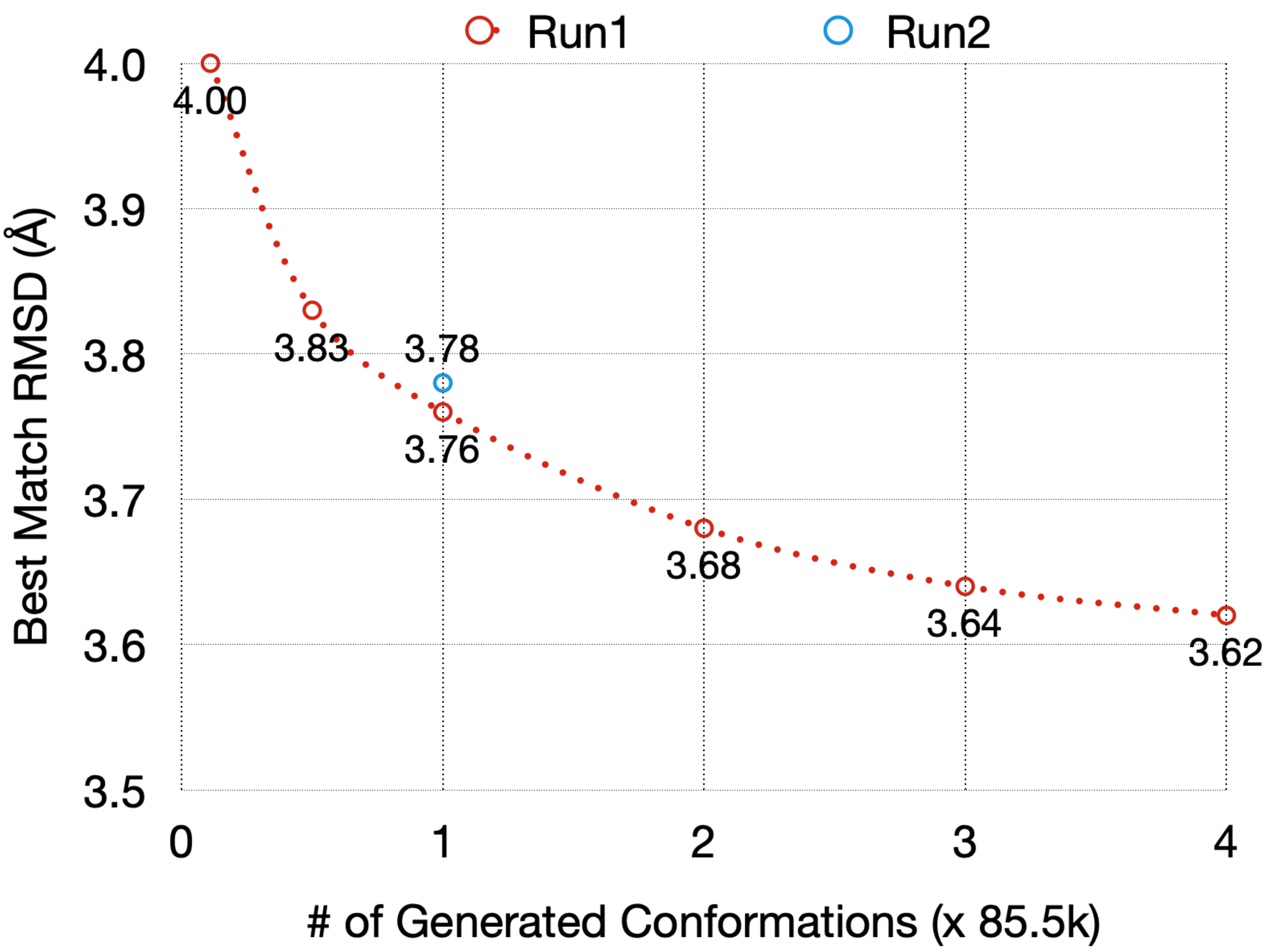

Figure S6. Results for autoencoder-generated conformations of Q15, with a 200-dimensioan latent space. The average best-match RMSDs of the 100-fold diluted test set are calculated against generated sets at different sizes. The latter sizes are measured in multiples of the test size (= 85,500). Run1 results are shown at sizes of the generated set ranging from the training size (9,500 or 0.11×) to 4×. For run2, the result is shown at 1×.

Supplementary References

1. Minh, D. D. L. Alchemical Grid Dock (AlGDock): Binding Free Energy Calculations between Flexible Ligands and Rigid Receptors. *J Comput Biol* **41**, 715-730 (2020).

2. Michaud-Agrawal, N., Denning, E. J., Woolf, T. B., Beckstein, O. MDAnalysis: A toolkit for the analysis of molecular dynamics simulations. *J Comput Chem* **32**, 2319-2327 (2011).

3. Hicks, A., Zhou, H. X. Temperature-induced collapse of a disordered peptide observed by three sampling methods in molecular dynamics simulations. *J Chem Phys* **149**, 072313 (2018).

4. Liu, P., Kim, B., Friesner, R. A., Berne, B. J. Replica exchange with solute tempering: A method for sampling biological systems in explicit water. *Proc Natl Acad Sci U S A* **102**, 13749-13754 (2005).

5. Terakawa, T., Kameda, T., Takada, S. On easy implementation of a variant of the replica exchange with solute tempering in GROMACS. *J Comput Chem* **32**, 1228-1234 (2011).
