# Supplementary material for: Artificial intelligence guided conformational mining of intrinsically disordered proteins
Source: Commun Biol. 2022 Jun 20;5:610. doi: 10.1038/s42003-022-03562-y (PMC9209487; doi:10.1038/s42003-022-03562-y)
Supplement: Supplementary file 4 — Description of Additional Supplementary Files [file 42003_2022_3562_MOESM4_ESM.pdf]

Description of Additional Supplementary Files

File Name: Supplementary Data 1

Description: The source data behind the graphs in the paper
